# Doctors as Resource Stewards? Translating High-Value, Cost-Conscious Care to the Consulting Room

**DOI:** 10.1007/s10728-022-00446-4

**Published:** 2022-05-13

**Authors:** Marjolein Moleman, Teun Zuiderent-Jerak, Marianne Lageweg, Gianni L. van den Braak, Tjerk Jan Schuitmaker-Warnaar

**Affiliations:** Athena Institute, Faculty of Science, VU University Amsterdam, De Boelelaan 1085, 1081 HV Amsterdam, The Netherlands

**Keywords:** Cost-conscious care, Health care costs, High-value care, High-value, cost-conscious care, Medical professionalism, Resource stewardship

## Abstract

After many policy attempts to tackle the persistent rise in the costs of health care, physicians are increasingly seen as potentially effective resource stewards. Frameworks including the quadruple aim, value-based health care and choosing wisely underline the importance of positive engagement of the health care workforce in reinventing the system–paving the way to real affordability by defining the right care. Current programmes focus on educating future doctors to provide ‘high-value, cost-conscious care’ (HVCCC), which proponents believe is the future of sustainable medical practice. Such programmes, which aim to extend population-level allocation concerns to interactions between an individual doctor and patient, have generated lively debates about the ethics of expanding doctors’ professional accountability. To empirically ground this discussion, we conducted a qualitative interview study to examine what happens when resource stewardship responsibilities are extended to the consulting room. Attempts to deliver HVCCC were found to involve inevitable trade-offs between benefits to the individual patient and (social) costs, medical uncertainty and efficiency, and between resource stewardship and trust. Physicians reconcile this by justifying good-value care in terms of what is in the best interest of individual patients–redefining the currency of value from monetary costs to a patient’s quality of life, and cost-conscious care as reflective medical practice. Micro-level resource stewardship thus becomes a matter of working reflexively and reducing wasteful forms of care, rather than of making difficult choices about resource allocation.

**Supplementary Information:**

The online version contains supplementary material available at 10.1007/s10728-022-00446-4.

## Introduction

Health care systems are facing various pressures for change towards more cost-effective and sustainable health services to ensure care for current and future generations [1]. Demographic changes have led to a shift in the burden of disease from infectious diseases towards predominantly lifestyle and ageing-related diseases, pushing health care systems to respond to a growing need and demand for health care. Combined with stagnant or decreasing resources being invested into health care globally, sustainable solutions are needed that enable all stakeholders involved to deliver high-quality care while keeping costs under control [2].

Policymakers around the world have tried various solutions from health budgeting, tackling fraud, enforcing clinical guidelines and making health care into a market-based commodity that turns patients into health care consumers [3–5]. Yet, none of these solutions has led to a durable transformation of health care systems [6]. A change of paradigm, often denoted as ‘from volume to value’ has marked a shift in the focus from cost control to improving value. In Box 1, we describe how this shift from ‘volume to value’ has taken shape in policy practices in the Netherlands.

In order to induce a shift from volume to value, we need to understand how costs relate to the quality of care [6, 7]. Burns & Pauly [6] illustrate this shift by contrasting the iron triangle with its successor: the triple aim strategy. The iron triangle argued that faced with constrained resources, societies must make trade-offs among three health care system goals: increased access, higher quality, and lower cost of care, which resulted primarily in strategies that focused on cost control. The framework perceived goals of cost reduction, access to health care and quality improvement as incommensurable [6]. The triple aim strategy, on the other hand, advocates that these three goals can be achieved simultaneously. It points to population health as the primary aim of health care systems, with the other two aims: reducing per capita health care costs and improving patient experiences as contributors to the primary aim [8]. In order to address the cost-quality issue and operationalize the triple aim, the authors point to two quality issues: the *overuse* of wasteful care and the *underuse* of helpful care that are affecting patients, providers, and health systems [9]. Thus, the pathway to *real* affordability, Saini et al. [10] note, is defining the ‘right care’.

**Box 1:** Transforming the health care system from volume to value–the case of the Netherlands

For well over a decade, policy initiatives in the Netherlands have been aimed at shifting policy efforts from cost control to improving value. In order to contain health care costs, the Dutch government adopted a system of regulated competition for hospital care in 2006 focused on improving efficiency while attempting to safeguard public values of quality and accessibility [11]. Although the government is formally responsible for protecting public values such as the accessibility, quality and affordability of care, ensuring the preservation of these values has largely been delegated to market actors including health care consumers (i.e. patients), health insurers and regulatory bodies, who are expected to act as quality- and cost-aware negotiators [12, 13]. Dutch citizens are allowed each year to select the insurance plan that best fits their health care needs [14]. In this aspect of regulated competition, patients were expected to act as the countervailing power that would force insurers, through selectively contracting providers, to compete in offering value for money for their clients
Effective resource stewardship, however, proved increasingly hard to achieve within the Dutch system of regulated competition, given that-to the dismay of the health economists and policymakers-consumer mobility has remained very low [15]. Furthermore, 90% of the insurance market is now occupied by four large companies [16], which further contests the idea of a ‘competitive’ market where citizens ‘shop around’ for insurance policies on the basis of value for money. This situation has generated issues of trust among actors [13, 17]. Health care providers and ‘consumers’ view health insurers as being too powerful, resulting in low levels of trust in fulfilling their assigned role to bargain for high quality of care [17]. An empirical analysis of market practices in Dutch health care conducted by Zuiderent-Jerak et al. [13] found that quality improvement programmes only seem of interest to health insurers if they reduce costs. Insurers are increasingly defining quality solely in terms of good outcomes at lower costs as they are merely seen as profit-oriented organisations if their premiums rise [13]. Within this arrangement, resource stewardship in relation to the cost/quality issue becomes merely a matter of cost-saving: insurance companies try to purchase acceptable levels of care at the lowest cost and those who buy insurance select policies at the lowest levels of premium

The triple aim frames quality and cost as factors dependent on the function and design of the system, rather than solely a function of the individual skills of the people that work in that system [18]. Affiliated frameworks including value-based health care and the quadruple aim do underline the importance of positive engagement of the health care workforce in reinventing the system in order to achieve the triple aim [19, 20]. Berwick [21] describes that this requires health care workers to take responsibility for system change and to acquire competencies “to set bold aims, measure progress, find alternative designs for work, and test changes rapidly and informatively” (p. i5) [21]. Notably, well-known initiatives including Choosing Wisely, Value-based health care and the ‘High-value care curriculum’ adopt a so-called ‘physician in the lead’ (PIL) approach [22, 23] that emphasises the role of (future) doctors as initiators of changes that promote resource stewardship, that is, despite some limitations of adopting a PIL approach, as opposed to a ‘team-led approach’ in the context of resource stewardship [23].

### Physicians as Stewards of Resource Stewards

The relationship between professional decisions and cost concerns is not new. Until recently, physicians justified their clinical decisions in terms of *personal value*, the incorporation of patient needs and preferences, and *technical value,* the incorporation of evidence-based health outcomes of individuals and populations [24, 25]. The third component of *allocative value*, on the other hand, brings considerations of individual patient needs and the needs of the entire population together and reconfigures professionals’ accountability from patient’s advocate towards balancing a duty to society with a duty to the individual patient [24].

Several authors promote the addition of a medical competency that teaches future doctors about resource stewardship [26–28], *i.e.* balancing the three values in medical practice. ‘High-value, cost-conscious care’ (HVCCC), proposed by Steven Weinberger [28] as “a seventh critical general competency”, has now been incorporated in several competency frameworks and educational programmes [29–33]. HVCCC is about preserving “the delivery of interventions that provide good value” by eliminating marginally effective health care and other sources of waste (‘low-value care’) and delivering health care that provides benefits that commensurate costs (‘high-value care’) [34].

Although several countries have adopted the idea of teaching (future) doctors the principles of HVCCC in order to embed resource stewardship in the medical profession, there are ongoing debates about the acceptability and feasibility of such micro-level resource stewardship. The incorporation of three aims, or three values (see also: The Triple Value Healthcare model), Storkholm et al. [18] and Martin et al. [35] note, has shown difficult to achieve in practice, as it forces together goals that traditionally have appealed to two competing logics: managerialism and professionalism [18, 36] and the idea that both are needed as countervailing powers [37]. Given the historical distribution of the responsibilities between managers and regulators on the one hand and professionals on the other, the controversy this caused is unsurprising. Debates revolve around either preserving the traditional patient–physician ethic or extending this to include resource stewardship [38–40]. Conceptual ambiguity further complicates this debate on medical ethics, especially with regard to whether making physicians responsible for resource stewardship inevitably entails bedside rationing-“restricting the use of any intervention, regardless of its effectiveness or value” [41]-or whether it offers an alternative to rationing. Some authors in these debates argue that the concept of HVCCC encourages physicians to commit to the notion of “rational care” that entails providing necessary, effective and efficient care for individual patients [26, 42]. This, they argue, would prevent the denial of medically necessary care to patients, a scenario that is often related to rationing. Such definitions of ‘rationality’ could be seen as attempts to help physicians assume their roles as resource stewards while avoiding a conflict with their current understandings of their professional role.

The lively debate on the ethics of shifting the roles of physicians notwithstanding, remarkably little attention is paid to what actually happens in the consulting room when doctors try to combine providing care that considers the needs of individual patients with a consideration of the needs of the entire population [43]. In order to establish an empirical grounding for discussions about resource stewardship, more insight is needed into how resource allocation decisions play out at the micro-level. How do physicians reconcile *allocative value* with their moral duty to provide care for individual patients? What tensions might their expanded professional accountabilities create when providing care to individual patients? And how could these tensions shape how HVCCC manifests in practice?

The objective of this study is to examine how physicians (in training) give meaning to resource stewardship in the context of their daily practice, what happens when resource stewardship becomes a ‘physician-in-the-lead’ approach and under which conditions such an approach could feasibly contribute to the overarching goal of contributing to the sustainability of the health care system.

## Methods

This study was conducted in teaching hospitals across the Netherlands that participated in the *Bewustzijnsproject*. The Bewustzijnsproject is a Dutch initiative, commissioned by the Dutch Board of Medical Specialists, that guided the implementation of HVCCC in postgraduate education in the Netherlands. The Bewustzijnsproject gathered existing HVCCC educational tools and initiatives, in order to assist fellowship programmes to develop HVCCC curricula. Among these educational approaches, the Bewustzijnsproject encouraged medical residents to set up new HVCCC innovative practices, referred to as ‘HVCCC projects’, which resulted in over 200 resident-led HVCCC projects.

### Interviews

We conducted semi-structured interviews with 49 respondents who were involved in initiating (residents, n = 39) or supervising (attendings, n = 10) so-called ‘HVCCC projects’ within the Bewustzijnsproject. Residents could set up a project to address issues they encounter in daily practice that hinder HVCCC delivery. A purposive sampling approach was adopted. In order to select relevant projects for this study, the research team screened the project plans of the HVCCC projects in order to select projects that addressed one or more components of HVCCC as defined by Owens et al. [34]: (1) a focus on the reduction of low-value care (LVC); (2) a focus on the promotion of good-value care (GVC); (3) a focus on raising cost-consciousness; and/or (4) a focus on the reduction of health care costs.

Five researchers, using a semi-structured interview approach with open-ended questions (Supplement), conducted the interviews. The interviews were either conducted or supervised by trained qualitative interviewers GLB and MM. In addition, three research interns, trained in qualitative interviewing during their master’s programme, conducted the interviews. Participants from 18 different hospitals were interviewed, with most respondents working at university medical centres (n = 35) and relatively fewer respondents working at local teaching hospitals (n = 14). The duration of the interviews was approximately 45 to 60 min.

First, we asked respondents to provide a general definition of HVCCC. It should be noted that although we refer to HVCCC as the central defining concept of this study, the Bewustzijnsproject phrased this as ‘doelmatigheid’-a balance between costs and quality of care, which in Dutch is often used synonymously with efficiency. After the first interview question, we directed the discussion to focus specifically on the concept of HVCCC as defined by Owens et al. [34]. We asked respondents to describe how they gave meaning to HVCCC through their project design choices. We guided the discussion about the HVCCC projects by asking respondents to indicate how the intended project outcome(s) could be plotted on the HVCCC matrix developed by the research team. This matrix delineates HVCCC into four dimensions based on the definition of HVCCC from Owens et al. [34].

The x-axis represents ‘quality of care’, comprising:

1. A focus on the reduction of low-value care, which indicates that projects intend to reduce unnecessary care and other sources of waste.
2. A focus on the promotion of good-value care, which indicates that projects intend to promote quality of care.
  The y-axis represents ‘costs of care’, comprising:
3. A focus on raising cost consciousness, reflecting projects that intend to improve physicians’ awareness of costs.
4. A focus on the reduction of health care costs, indicating that projects intend to lower costs of the care provided.

We encouraged respondents to explain in their own words how their project fitted within the quadrants of the HVCCC matrix. We deliberately chose to use the matrix as a tool to first discuss the envisaged project outcomes, in order to clarify the distinction between intentions (goals) and practice (implementation) and allow for a reflection to explain potential differences between the two. Thus, besides discussing the respondent’s intended conceptualisation of HVCCC, the interviews explored whether and how HVCCC may have been further shaped during the process of implementation. During the data collection phase, the researchers held regular meetings to discuss findings and identify attention points for follow-up interviews and triangulate findings (see also Table 1). Using the insights derived from the interviews, we sought to uncover how conceptual, practical, and cultural challenges shaped HVCCC considerations in medical practice in order to reflect on its implications on doctors’ professional accountability and on HVCCC as a concept for sustainable medical practice. The research activities regarding data collection and data analysis are summarised in Table 1.

**Table 1:** Methods and related research activities

Data collection and data analysis	Research activities
Interviews	Definitions of HVCCC
	Envisioned projects outcomes depicted on the HVCCC matrix—probing questions were used to elicit how respondents gave meaning to the dimensions of HVCCC through their project design choices.
	Implementation of HVCCC—probing questions were used to elicit conceptual, practical and cultural challenges that shaped HVCCC in use.
	Triangulation of findings—respondents were asked to reflect on challenges reported by other respondents when similar topics came up during the interview.
Member check	All the interviews were summarised and sent to the respondent for member check. Respondents were asked to supply changes and additions via email. Summaries and subsequent analyses were adjusted accordingly.
Thematic analysis	Interviews were audio-recorded and described verbatim by the principal interviewer.
	Inductive codes were assigned to the interview data by the principal interviewer.
	Inductive codes were paired with labels: espoused HVCCC, HVCCC in use, in addition to inductive codes to identify factors that could explain discrepancies between the two.
Thematic synthesis (I)	***From espoused HVCCC to HVCCC in use:***
	MM and GB clustered patterns regarding espoused HVCCC, *i.e.,* how do physicians (in training) envision HVCCC as a practice.
	MM and GB clustered patterns regarding HVCCC in use, *i.e.,* what does HVCCC become in the context of physicians' daily work.
	MM and GB clustered patterns regarding discrepancies between espoused HVCC and HVCCC in use.
Thematic synthesis (II)	During a consensus meeting MM and GB discussed discrepancies between *espoused HVCCC* and *HVCCC in use* together with the research interns who were involved in data collection and decided upon the final categories describing the conceptual, cultural and practical challenges that shape HVCCC in practice.
Validation of study findings	Findings were discussed during six reflection sessions attended by the project commissioners and educationalists involved in the Bewustzijnsproject to collect feedback and validate the findings.

### Data Analysis

Each interview was audio-recorded and transcribed verbatim. All the interviews were summarised and sent to the respondent for member check. Respondents were asked to supply changes and additions via email. Summaries and subsequent analyses were adjusted accordingly. Recruitment, data collection, thematic analysis were concurrent activities. Each interview transcript was analysed by the principal interviewer who coded inductively to elicit themes present in the HVCCC definitions and their operationalisation as reflected in the HVCCC projects. The analysis focused on uncovering how physicians conceptualised HVCCC (‘espoused theories’) and how HVCCC is translated to the consulting room (‘theories in use’). We used the concepts of espoused theories and theories in use, developed by Argyris and Schon [44], to guide the analysis of what respondents believe guides HVCCC provision (espoused theories), and what HVCCC actually becomes in practice (theories in use). During the analysis process, the researchers met regularly to develop an iterative and shared conceptual understanding of the data. Finally, six reflection sessions with the project commissioners and educationalists involved in the Bewustzijnsproject were organised to collect feedback from experts in the field of medical education and to validate the findings.

## Results

This section outlines physicians’ conceptualisations of high-value, cost-conscious care and compares these ‘espoused notions’ of HVCCC with its actual translation into the consulting room (‘theories in use’). In the remainder of the results section, we describe our analysis of the differences between respondents’ conceptualisations of HVCCC (espoused HVCCC) and what HVCCC actually becomes in practice (HVCCC in use).

### HVCCC Matrix

**Fig. 1:**
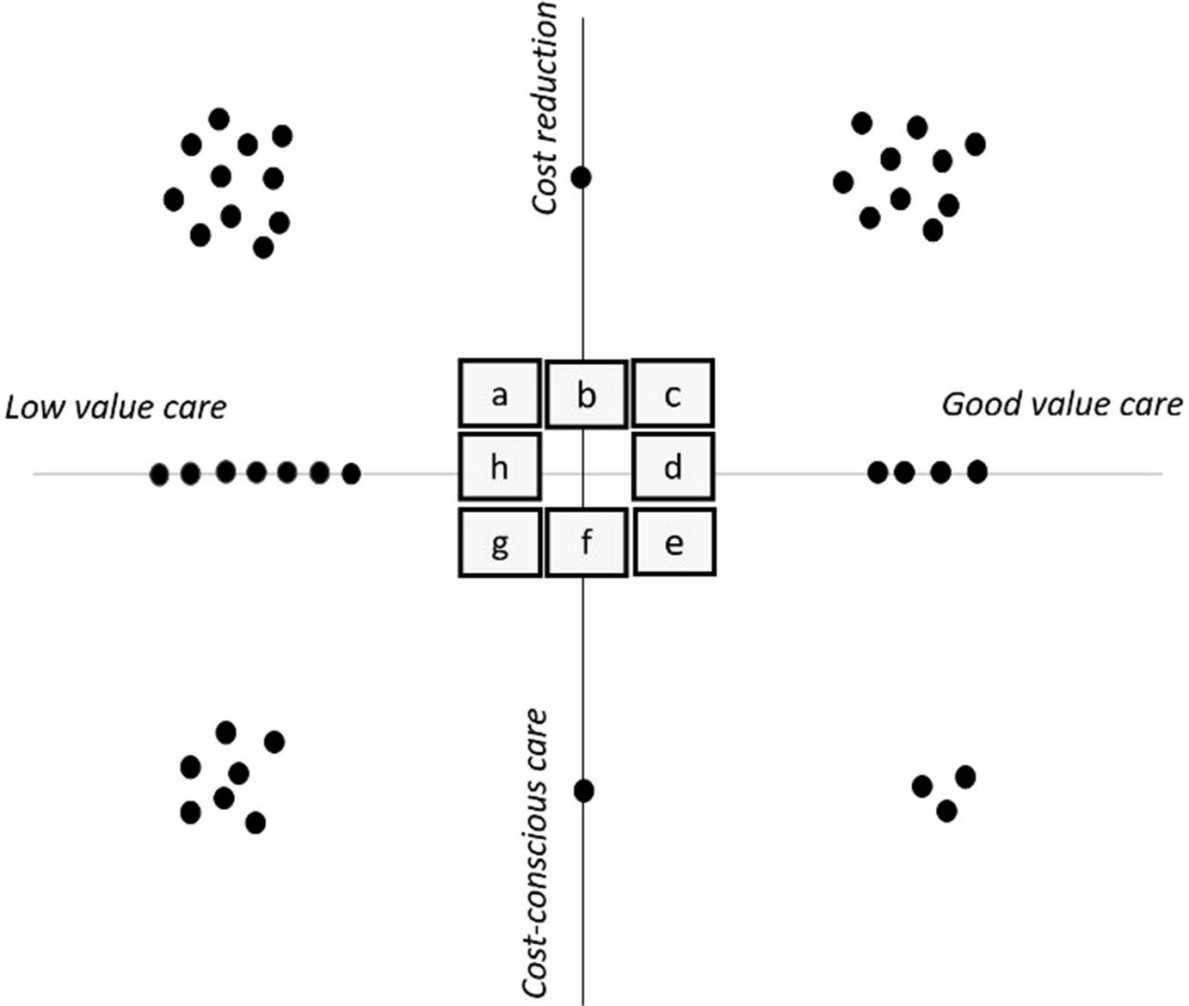
Espoused HVCCC

**Fig. 2:**
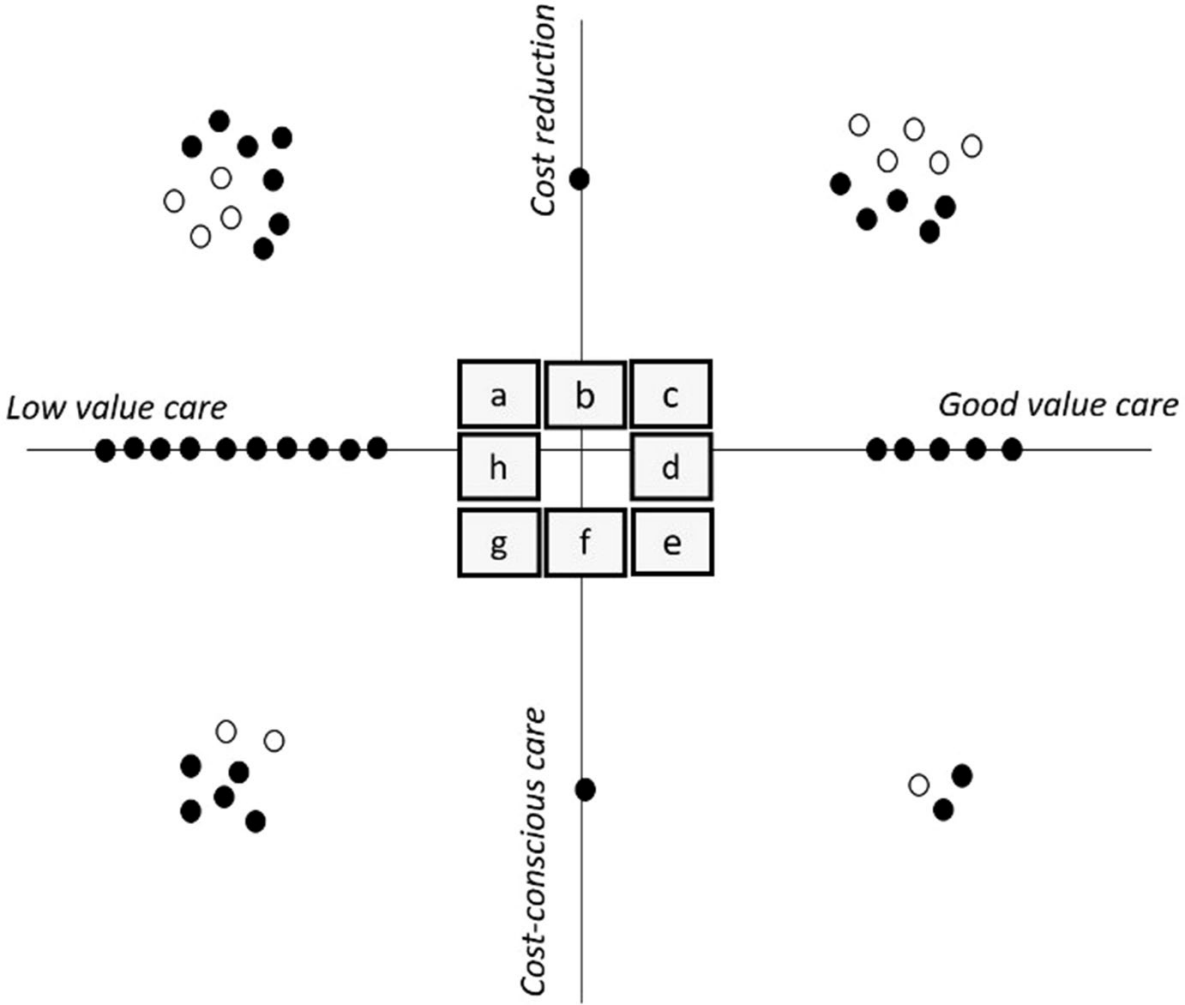
HVCCC in use

In order to examine how respondents conceptualise HVCCC (espoused HVCCC), we asked respondents to plot envisaged HVCCC project outcomes on the ‘HVCCC matrix’. Figure 1 provides an overview of the envisaged aims of respondents’ HVCCC projects. Respondents were encouraged to explain their choices in order to gain insight into how envisaged ideas of HVCCC (‘espoused theories’) were actually translated into practice. These discussions revealed that physicians (in training) encounter several complexities in medical practice that shape how HVCCC manifests in practice. Figure 2 shows differences between *espoused HVCCC* delivery and *HVCCC in use,* which are further explicated in Table 2.

**Table 2:** Overview of quotes illustrating the differences between espoused HVCCC and HVCCC in use

Aspects of HVCCC as defined by Owens et al. (34)	Espoused	In use
1. Raising cost-consciousness	**In order to use health care resources efficiently (and equitably), one should be aware of its (comparative) costs.** *A substantial part of health care is spent on intensive care and I think it is very important that future doctors know that the care that they deliver is very expensive and that we have to try to reserve care for those people that are most likely to benefit from it (..) in order to make those decisions, we need to know the outcomes of care and something about the costs* (P1)	**Framing cost-conscious care in terms of ‘costs for the patient’.** *[In our app] we did not use monetary costs, but framed it in terms of ‘costs for the patient’ (..) ‘is this test maybe a bit too invasive considering the patient’s condition?’, ‘does this have a lot of potential side effects?’ (..) In my opinion, that is more important than money, even though promoting effective, efficient health care could definitely result in cost reductions, the starting point is not: ‘we should discharge this patient as soon as possible [to reduce costs]’, but instead: ‘how can we switch from IV to tablets as soon as possible, to prevent infection, loss of mobility and loss of muscle mass’* (MR 3)
2. Reduction of health care costs	**Cost reduction is a secondary and desirable by-product of eliminating wasteful care, selecting the cheapest option when interventions offer comparable quality or promoting helpful care.** *Endometriosis is a multidisciplinary disease, its treatment often very costly [because of diagnostic delay]. If given the right recommendations, the GP could prevent patient harm and need for extensive treatment and this could eventually reduce costs of care as a result. (MR 26)* *Teaching residents to critically assess the necessity of a lab test: ‘why do I want to order this test?’. This critical reflection is expected to reduce unnecessary testing and decrease health care costs due to a decrease in the total number of lab orders (MR 2)*	**Cost reduction is not my professional intention. Patient experience trumps or even replaces cost reduction.** *I think you should leave costs out of it. First, and foremost, it is about optimising care for patients and yes, that could indirectly lead to cost reduction. It is absolutely not my intention, but it could lead to a reduction in costs. (MR 14)* *My motivation is not so much to reduce costs, which of course is a very important factor considering the need to cut costs. But [cost reduction] should not go at the expense of something else, but should only [allowed] in favour of the patient, [in the sense of] reducing harm.* *It is not at all about costs It is about preventing complications. It is not so much about cost-consciousness. Of course, you save money when you prevent complications, it reduces the length of hospital stay, but it is not my intention to reduce costs.*
3. Reduction of low-value care	**The delivery of care in which benefits and harms commensurate the costs (good-value care) can be achieved by improving the efficiency of care (low-value care ↓), without lowering the quality** *Delivering high-quality care with minimal use of resources. (..) That is my first impression of HVCCC. (..) I do not think my project relates to HVCCC, because I have not considered costs at all.*	**Low-value care ↓ patient value ↑** *[We need to make sure that a patient is brought to an emergency room that has sufficient capacity] If the patient comes to an emergency room where there is sufficient time to make the right diagnosis, this improves the quality of care. Unnecessary care could be prevented as well as possible misdiagnoses (..)* *Research shows that administering more blood tests also has negative consequences for the patient. I am convinced that doing fewer blood tests improves care.* *The [app] helps to reduce complications during surgeries.* **Low-value care is about not doing ‘too much’** *The primary goal is to deliver the right care to the right patient added with considerations of costs and quality (..) Once [a patient] enters the hospital circuit, they are susceptible to overdiagnosis (..) all tests and treatments have side effects. [Internists] have a different perspective: a hospital perspective. In academic hospitals, we see very rare conditions, [it is] a whole different epidemiology compared to primary care. When patients complain about hip pain, a GP would recommend patients to take some rest and call back in one week. But when patients bring up hip pain when they just happen to visit their internist [for something else] the internist may immediately order a scan, request an orthopaedic consult, and maybe even refer the patient to the neurologist. All caused by differences in perspective and yes, that is also very expensive and sometimes just ‘too much’. (..) Clinical practice guidelines may be very helpful to decide where the patient actually belongs.*
4. Promotion of good-value care	**Quality of care ↑ Low-value care ↓** *If you deliver high-quality care then you are using your resources efficiently.* *Delivering optimal patient care, if possible, at a place that is closest to home.*	**Optimising quality of care that improves patient experience and/or outcomes of care** *The goal of HVCCC, in my opinion, is pursuing the goal of the patient, which is to have a good quality of life (..) refraining from delivering care that does not promote that goal (..) [reducing costs in this context means] minimising the harm done to the patient (..) delivering care that you expect will benefit the patient, guided by what the patient values (..) by discussing patient’s wishes, we can avoid that patients are admitted to the ICU, contrary to their wishes (..) we [open up space] to choose not to intervene.* **Attuning clinical practice guidelines to patient value** *Cost-effectiveness also has a lot of ifs and buts, for example, because we cannot always measure effectiveness adequately (..) Now, we are talking more about experienced quality [of care]. Sometimes you see a one-millilitre reduction in blood loss or something. But does that one millilitre matter? Does the patient suffer from that? [Outcome measures should be medically relevant, not just statistically significant].* *You do not know how others look at life.. and as doctors, we should think less in medical terms (..) that is very much our agenda [our default] but the parents [of newborns] also have an agenda (..) The clinical guideline prescribes counselling, but it does not say how (..) I want to reduce practice variation in counselling [by adjusting the guideline] (..) I want to support parents to make a decision that aligns with their norms and values (..) variation based on the opinion of a particular doctor, that is unwarranted practice variation.*

Both Figs. 1 and  2 display very few projects related to cost-conscious care. The espoused notion of cost-conscious care (details in Table 2) associates cost-aware care with the need to reserve expensive and scarce resources for patients that are most likely to benefit from them. Notably, only five respondents, all of whom work in a setting of (looming) resource scarcity such as intensive care units, linked HVCCC to what closely resembles the notion of *allocative value* as proposed in The Triple Value Healthcare model [see e.g., 24] in which individual patient care is reflected on in light of a larger population of patients. Findings showed that applied in practice (in use), *cost-aware care* takes on a very different meaning. In Fig. 2 this difference is most striking in quadrants a and c: where a significant number of projects ‘disappear’. Whereas espoused cost reduction acknowledges the possibility of cost reduction as resulting from eliminating wasteful care, for example*, cost reduction in use* rejects the notion of cost reduction altogether. Instead, it offers ‘costs for the patient’ as an alternative currency in the value equation and emphasises patient experience as the primary goal of HVCCC delivery (details in Table 2).

Both Figs. 1 and 2 show that the *reduction of low-value care* (LVC) is more strongly associated with the concept of HVCCC compared to *the promotion of good-value care (GVC),* which suggests that *quality of care* was mainly defined in terms of ‘reducing LVC. Espoused LVC reduction denotes that efficiency of care is improved, without lowering the quality of care. However, in use, physicians (in training) add an additional justification to the elimination of waste, namely that patient value, and thus the quality of care, is improved as a result (Table 2). In use, the elimination of LVC thus shows considerable overlap with the promotion of GVC, in that improved efficiency contributes to a favourable balance between benefits, harms and costs of care (LVC ↓ →  GVC ↑). According to respondents, espoused promotion of *GVC* denotes that improved quality of care leads to, or even implies that, resources are used more efficiently (GVC ↑→ LVC ↓). In use, the perspective on promoting GVC extends to tools such as clinical practice guidelines, thereby linking two values from the Triple Value Healthcare model: personal value and technical value.

In the next paragraphs, the study findings are discussed in further detail. We reflect on the differences between *espoused HVCCC and HVCCC in use*, starting with costs (the y-axis of the HVCCC matrix), followed by a reflection on quality (the x-axis of the HVCCC matrix) and their implications on the delivery of HVCCC in practice.

### The Costs-Axis

In this paragraph, we reflect on (1) the virtual absence of projects that promote cost-consciousness and on (2) the exclusion of monetary costs from the value equation by rejecting costs as an *intentional* outcome of HVCCC considerations (in use).

‘Cost-conscious care’ comes down to the question of what physicians should ‘do’ with information on costs in clinical practice. The few projects (n = 11) that promoted cost-consciousness were educational projects that offered courses on costs to medical students or trainee doctors, as opposed to HVCCC projects directly related to patient care. *Espoused cost-conscious care*, as depicted in Table 2, relates the need for cost-conscious care to resource scarcity. Findings showed that *cost*-conscious care was almost exclusively mentioned by physicians (in training) that worked in settings of (looming) scarcity, such as the intensive care unit, and the resultant need for resource prioritisation. The majority of respondents, however, did not link cost-conscious care to ‘allocative value’. A medical resident, for example, notes that: *“we do display costs of care [in our project], but we do not want to scare people off in the sense that they start ordering too little tests, because underordering also comes at a cost”*. Table 2 shows that ‘in use’, most respondents emphasise considerations of personal value, *i.e.* ‘what are the patient’s needs, preferences and goals?’ and technical value: *i.e.* ‘is this treatment clinically indicated?’, in order to ensure the right care for individual patients. Whereas costs can indeed provide an incentive to critically assess the need for medical intervention: ‘why do I want to order this test?’, it serves more as an invitation to reflect on the personal and technical value but rarely about balancing individual and population-level values.

Table 2 shows that with regard to ‘espoused cost reduction’, respondents generally expect cost reductions as a result of tackling overuse of wasteful care or underuse of helpful care. Nevertheless, when translated to practice (‘cost reduction in use’) several physicians (in training) distanced themselves from cost control, noting that reducing costs sits uncomfortably with their professional intention to offer the best care for individual patients. Some respondents experienced difficulty with cost reduction as an outcome measure because it would imply that costlier care is frowned upon: *“Patients always cost money, sometimes you need to opt for costlier tests in order to catch a diagnosis more quickly*” (MR 3). An attending, among others, prefers the notion of: *“expensive [care] when necessary, cheaper when possible”.* Although this keeps the focus on individual patient care, the question of ‘how expensive is ‘too expensive’?’ forces trade-offs between individual patient care and population concerns back into the picture. Notably, this trade-off was only brought up by a few respondents. They noted that the ethical question of: “what is good value care worth?”, is a public and political debate, not a decision that physicians can or should want to make in determining patient care since it could go at the expense of putting patient’s welfare first. An attending physician, for example, asked, “*Who are we [as physicians] to refuse admission to the ICU, because it is too expensive?”* (P1). One respondent related physicians’ struggle with providing cost-aware care to the absence of politically defined limits to guide cost-conscious care in practice, impairing judgements as being ‘too expensive’ or ‘not justifying the costs’. This indicates a tension between the government being formally responsible for safeguarding the affordability of the health system but delegating its operationalisation [13] to, in this case, physicians.

A couple of respondents mentioned the risk of trust issues. In previous resource stewardship arrangements (see also Box 1), the responsibility was focused on health insurers who primarily defined resource stewardship solely in terms of good outcomes for less money [13]. This predominant focus on cost-control generated public trust issues towards health insurers. Similarly, physicians and hospital organisations try to avoid distrust caused by the impression that physicians consider costs in providing health care, as illustrated in the quote below.

We placed an order at the hospital’s communication department to have pocket maps that displayed health care costs plasticised. But the communication department did not agree with the distribution of these pocket maps as it would project an image that our department is focused on money and that we would refrain from delivering care because of it (..) They were very critical about the intentions of the pocket maps and the risk of negative publicity (MR 16) (..)

In this section, we established that cost-consciousness may act as an incentive to reflect on personal and technical value. Given the tensions of cost considerations from within as well as outside the medical profession, respondents sought other ways of (implicitly) weighing and balancing the different values. In the next section, we analyse how this balancing act is reflected in strategies to ‘reduce low-value care’ and ‘promote good-value care’.

### The Quality-Axis

First, we discuss the most prevalent strategy related to the quality of care, namely: reducing LVC. We describe common *in use LVC* strategies and reflect on the challenges related to them. We then expand on the connection between LVC and GVC, in other words: does the reduction of LVC improve GVC? Finally, we describe research findings in relation to GVC promotion and use insights to foster connections between GVC and LVC.

#### Low-Value Care

One of the most common definitions of HVCCC provided by respondents was: ‘doing the same, with less’, in other words: using less resources to achieve the same quality result. The data showed two dominant approaches: 1) reflective decision-making, and 2) adherence to clinical practice guidelines (CPGs).

According to many respondents, deliberate reflection on the medical necessity and appropriateness of care is fundamental to providing appropriate, high-value, cost-conscious care. Continuously reflecting on need: “what does this patient need?”, “does this test change management?” can contribute to more responsible use of resources, because it is said to help to reduce unnecessary tests, risks and thus promote patient-friendly care, as noted by a resident, “*If I eliminate unnecessary blood tests, it lowers the number of times I have to stick the patient with a needle and it lowers the risk of complications such as phlebitis”* (MR 2). CPGs could aid this process of reflecting on the need for medical intervention. Three projects used CPGs to assess whether the right patients were being treated in the right place. Combined with an infrastructure that improved transmural communication, these projects envisioned treating eligible patients closer to home informed by CPGs. On the one hand, this can be related to the notion of personal value by improving patient experience (e.g., eliminating unnecessary consultations, reducing travelling time, familiar general practitioner) and preventing avoidable harm. As one resident noted: “*once patients enter the hospital circuit, they are susceptible to overdiagnosis (..) every test and treatment has side effects”.* On the other hand, ‘treating the right patients in the right place’ helps reserve hospital services for patients for whom secondary or tertiary care is clinically indicated (allocative value).

In this context, all of the three values from the triple value framework are involved in HVCCC delivery. Nevertheless, study findings uncovered several dilemmas of delivering HVCCC in the so called ‘grey zone’-when there is scientific uncertainty about the ‘best care’ for individual patients [see also: 10,44]. Based on our analysis, we identified three inherent characteristics of medical practice: medical uncertainty, treatment of individual patients and the diagnosis of symptoms, which raise questions about the efficient application of CPGs and may generate discrepancies between *espoused elimination of LVC* and *LVC elimination in use*.

##### Medical Uncertainty

CPGs offer a tool to provide efficient care, as one resident noted: “[The guideline taught me] *not to opt for an abdominal x-ray in order to diagnose kidney stones. (..) [because] the imaging is often blocked by patients’ bowel movement (..) So that is totally inefficient*” (MR 22). In this situation, the efficient choice seems obvious. Nevertheless, the efficiency of ordering test X to diagnose disease Y, as prescribed by the CPGs, is challenged by uncertainty-the efficiency of the clinical decision made can only be established in hindsight after test results have come in. Hence, justification of medical choices takes the form of the most reasonable gamble possible. Tensions arise from trying to manage uncertainty while at the same time trying to provide appropriate and efficient care, as illustrated in the quote below.

I tried to figure out why one of my patients had blood in his urine. We did multiple tests, but we could not figure out the cause. After one year, I wanted to discharge the patient, but my boss suggested doing one last scan, just to be sure. When the scan results came in, it turned out that the patient had developed a large kidney tumour over the past year. This tumour was not detectable on the scan we did the year before (MR 22).

The quote illustrates the challenge of providing efficient care in a context characterised by uncertainty. When the influence of medical uncertainty on clinical decisions becomes excessive, the resulting defensive medicine can defeat the purpose of HVCCC. Then again, whether a medical decision is *defensive* or *justified* can only be established after the event.

##### From Population-Defined Risks to Individual Patients

The uncertainty of applying CPGs to practice is also related to the translation of population-level data that underpins CPGs to individual patient cases, each with its unique set of variables. “*Patients are not interested in a leaflet that refers to some scientific article that says: ‘Our study of 100 patients showed that the chances of X are 3.8%’. Patients just want to know: ‘does it apply to me or not?’”* (MR 30). This respondent illustrates the challenge of determining proper treatment to meet the needs of individual patients based on information about risks and preferred treatment defined on a study at the population level. A resident noted that the average length of CPGs militates against the efficient use of these tools in practice: “*[The protocol for treating pneumonia] is 130 pages. That covers pretty much 100% of patients encountered in practice, including all exceptional cases and contra-indications, but [all this extra information] renders the protocol rather illegible”* (MR 35). The tendency of CPGs to cover as much ground as possible, factoring in all possible scenarios^1^ to represent most patients encountered in practice, at the same time undermines the feasibility of using them in a time-constrained working environment.

##### Diagnosing Symptoms or Diseases?

The previous paragraph described the problem of the CPGs’ ‘lengthiness’. This, added to their disease-specific nature, could further complicate efficient HVCCC considerations. Clinical guidelines are categorised by diseases, whereas in practice patients present a cluster of symptoms rather than a readily diagnosed disease, as outlined in the quote below.

There is a guideline for Irritable Bowel Syndrome, in which the possibility of endometriosis is mentioned only once in a tiny footnote. However, other than that there is no guideline for diagnosing endometriosis, or even for menstrual complaints in general, [one of the] red flags for endometriosis. If there were a guideline for these symptoms that could indicate endometriosis, this would increase GPs’ awareness and [shorten diagnostic delay]. (MR 9)

The quote illustrates tensions between the way that the CPGs categorise by diseases and patients who simply do not present with a readily diagnosed disease, which can cause significant delays in diagnosis, especially for under-recognised conditions.

The three practice complexities challenge the notion that using technical value to reduce low-value care always promotes personal value. Although this may seem obvious, these findings are illustrative of the difference between strategies to reduce waste and strategies to promote patient value. Whereas LVC is about doing less, while *maintaining* patient value, GVC is about optimising patient value first and delivering resources accordingly. In the next paragraphs, we analyse how GVC projects can reframe tensions related to cost-conscious care, cost reduction, and the role of CPGs, and what opportunities emerge for balancing of the triple values in the clinical encounter.

#### Good-Value Care, or: Putting Patient Value First

The first tension that we described in the results section is the consideration of costs in the delivery of HVCCC. Although many respondents considered cost-consciousness an incentive to deliver HVCCC, they generally discarded costs as part of the intention of HVCCC and thus removed it from the value equation. Question is, whether the three values are being weighed when costs are not part of this value equation, and instead, only quality is being considered. When physicians opt for an exclusive focus on quality, they evade part of the tripe aim strategy, that of combining cost control with patient value, in order to promote population health. A resident noted that “*colleagues generally only focus on content and quality, which often leads them to order more therapy”* (MR 36). The resident concluded that without cost-awareness and reflecting on value in relation to costs, it cannot be ascertained that strategies that improve quality are also more cost-effective than existing alternatives. A more nuanced perspective on this issue is provided by a couple of residents who noted that projects that combine the promotion of patient value with the reduction of low-value care can have an impact on resource utilisation. These projects attempt to avoid delivering ‘too much care’ from the perspective of the patient’s well-being. The pros and cons of intervening or doing nothing are evaluated in light of patients’ values and wishes, the impact on their quality of life and so forth.

The most important thing is creating more awareness, in the sense that everything that can be done, should not necessarily be done. Making more deliberate choices about resuscitation, discussing more openly with patients to make sound decisions on whether or not to pursue emergency life support based on data and discussions with patients about what value and quality of life mean for them (..) as doctors, we should not make matters for patients with already poor quality of life worse by resuscitating them (MR 21).

Respondents noted that there should be more emphasis on what care ‘costs’ patients in terms of their quality of life. Redefining the value currency from financial costs to refer to a patient’s quality of life (see also Table 2: ‘cost-consciousness in use’) underlines the idea that conversations about therapeutic preferences and restrictions are intended to minimise patients’ suffering, not in the interest of reducing financial costs.^2^

Although initiators of such projects emphasised the inclusion of patients’ perspectives on value, tensions between resource stewardship and acting in patients’ best interest persisted, especially in discussions of putting quality of life before longevity. Fear and resistance, partly fed by a larger social denial of the reality of human mortality, can invalidate physicians’ efforts to provide care in the patient’s best interest. Conversations about resuscitation, for example, can quickly be misinterpreted as doctors giving up on their patients.

You can imagine that optimising medications has an effect in the sense that it improves patients’ quality of life, and eventually a reduction of costs. And [the latter] is not important on a patient level, but it is on the population level. Every time I brought up this cost aspect during presentations, a couple of physicians, (..) generally five to ten per cent, would resist (..) saying: ‘Gee, it is not about costs, right? It is about the patient (MR 14).

It is a societal issue; people avoiding conservations about death (..) Recently, a couple of newspaper articles were published, stating that physicians should not bring up resuscitation when patients have just been submitted to the ER, because that could cause significant distress in patients, but this depends on how this conversation is held (MR 26).

What the three quotes have in common is the importance of honest conversation and shared decision-making to elicit patients’ values. In order to support such decisions, five projects explored ways of ‘attuning CPGs with patient value’ (see also: Table 2). One project, for example, was specifically devoted to the development of conversation guidelines (to be included as an appendix in the CPG), in order to support value-based care for patients. Other projects emphasised the importance of outcome measures that matter most to patients, such as quality of life one year after resuscitation.

In the discussion, we reflect on the strategies in light of the triple values: personal, technical and allocative value involved in HVCCC delivery and formulate directions for further research that builds on the notion of avoiding patient harm as a guiding principle for micro-level resource stewardship.

## Discussion

**Fig. 3:**
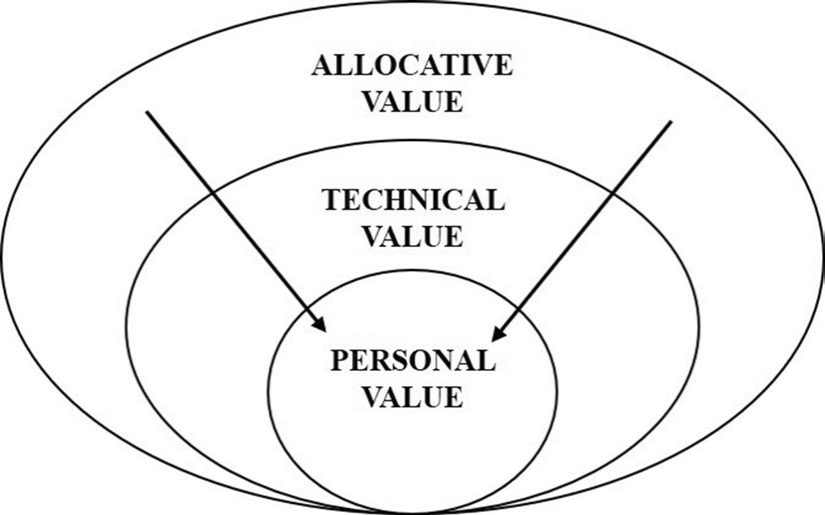
Fears of bedside rationing. The arrows pointing inwards represent concerns of ‘bedside rationing’, cost control through restricting the use of any intervention, regardless of its effectiveness or value. It represents concerns that allocative value displaces professional goals to put patient’s welfare first. Study findings suggest that arrows only direct inwards: from allocative value to personal value, when physicians are confronted with immediate resource scarcity. In this setting, scarce resources are prioritised for patient most likely to benefit from care

**Fig. 4:**
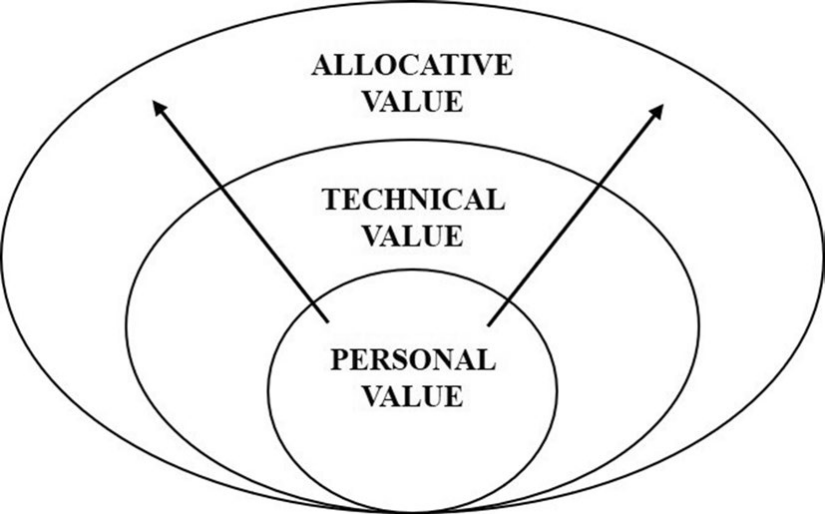
Balancing the triple values in resource stewardship. The arrows pointing outwards represent conditions that are conducive to HVCCC delivery. The model starts at the inner level (1) Identifying personal value: i.e. what are the patients needs and wishes, and avoiding doing more harm than good, which informs the second value (2) technical value, i.e. what outcome measures matter most to patients? and including such outcome measures in CPGs, and (3) utilising (or allocating) resources accordingly and reserving them for patients that are expected to benefit from care

This study explored what happens when resource stewardship is extended to the consulting room and under which conditions such a micro-level approach to resource stewardship could feasibly contribute to the overarching goal of contributing to the sustainability of the health system. Proponents of a physician-in-the-lead approach for resource stewardship argue that physicians are qualified to guide resource steward efforts because they have a better understanding of health-related decisions and their consequences compared to non-clinical actors [46]. Furthermore, Cooke [26] and Hood & Weinberger [42] note that the allocation of resources guided by physicians would lead to a better balancing of the triple values since physicians also fulfil the role of patients’ advocates. Still, concerns regarding the tensions that a duty to promote resource stewardship can create for the traditional care ethic [39, 40, 43] and fears that efficiency displaces professional goals such that professionalism becomes tainted by association [35] call for closer examination of how resource-allocation decisions are actually made at the micro-level. In this section, we reflect on study findings that resonate with these concerns and highlight the need for conditions that support micro-level resource stewardship that help shift the focus from directing inwards to directing outwards, as illustrated in Figs. 3 and 4.

### The Feasibility and Acceptability of ‘Physician in the Lead’ Resource Stewardship

In this section, we reflect on the feasibility and acceptability of HVCCC as a ‘physician-in the lead’ approach to resource stewardship. First, findings show that physician-in-the-lead resource stewardship does not align with the notion of *‘rational care’* as suggested by authors of seminal papers on HVCCC as a ‘seventh critical competency’ [28, 34]. This study found that physicians encounter several complexities in practice, including medical uncertainty, the treatment of individual patients rather than basing clinical judgements on population-level data, and diagnosis of symptoms rather than uncomplicated diseases, which contradict the notion of a straightforward balancing act of benefits, harms and costs. Proponents of HVCCC consider this balancing act as a process that ‘rationally’ paves the way towards optimally efficient and effective health care. In practice, HVCCC involves many trade-offs-in which improving one aspect will most likely have an adverse effect on another. Several examples of such negative correlations between quality and efficiency were found in this study. One example described in this article was medical uncertainty. As uncertainty is inherent in medical practice, physicians can only establish whether resources were used efficiently or defensively after the fact, which resonates with scholarship on the complexities of diagnostic work [47]. Other authors corroborate that medical uncertainty poses barriers to the provision of cost-aware care [41, 48]. Methods proposed to deal with uncertainty, such as CPGs and reflective decision-making, are not exempt from these quality–efficiency trade-offs. Reflecting on *each* medical decision as a means to use resources responsibly sits uncomfortably with the required investment of time to continuously reflect on each decision made, which speaks to the need to frame reflexivity rather than promote it in health improvement [49]. Whereas authors such as Owens et al. [34] and Reuben & Cassel [50] propose that HVCCC paired with adherence to evidence-based CPGs and measurement of health care outcomes would lead to the rational provision of care, this ‘rationality’ of medical practice can often only be established in hindsight, which implies that these ‘rational’ tools offer limited solutions to promote value in the clinical encounter. These findings highlight the relevance of conducting further research on including medical uncertainty in medical education, as proposed by Tonelli & Usphur [51].

Second, study findings showed that the ethical acceptability of shifting resource stewardship to the consulting room was a recurring topic, particularly with regard to physicians’ role in reducing health care costs. This study found that physicians distance themselves from the *intention* to reduce costs in providing care. Although the possibility of reducing costs is generally acknowledged, cost reduction is not the goal of achieving HVCCC from the doctor’s perspective. This study showed that delegating resource stewardship responsibilities to physicians puts them in an awkward position between acting as patient’s advocates and promoting allocative efficiency at a broader population level. In practice, this plays out strongly in favour of acting in the patient’s best interest. This resonates with empirical work from van Delden et al. [52] who found that physicians only consider allocation decisions just when they are in the best interest of the individual patient and a study conducted by Hurst et al. [43] that showed that few physicians use ‘distributive justice’ to guide medical decision making when confronted with limited resources, but often make such decisions in deliberation with others. This also resonates with suggestions made by Malik et al. [23] to expand the focus on a ‘team-in-the-lead approach’. We conclude that although study findings do not resonate with conceptual definitions of *rational care*, they do resonate with conceptual definitions of ‘right care’, particularly regarding current proposals by Brownlee & Korenstein [53] who suggested that framing overuse as potential harm engages providers by appealing to their professionalism and commitment to care for each individual patient and “do not harm”.

## Conclusion

This study contributes to the debate about what happens when responsibility for resource stewardship is extended to the micro-level to include physicians as potentially effective resource stewards. Study findings point to a couple of strategies that could help make conditions more conducive to ‘micro-level resource stewardship’. What these strategies have in common is that they focus on avoiding potential harm to individual patients. Moreover, such initiatives can be seen as attempts to align the triple values: personal, technical and allocative value, for example, by attuning clinical practice guidelines to patient value. This study points to further exploration of the conditions that support a framing of overuse as potential harm to individual patients that appeals to doctors’ professionalism.

## Supplementary Information

Below is the link to the electronic supplementary material.

Supplementary file1 (PDF 86 kb)

## Data Availability

The anonymised data and materials of this study are available from the corresponding author on reasonable request.

## Footnotes

1 Taking into consideration that multi-morbidity is only partially included in clinical guidelines, which can reduce the quality and safety of care provided to patients with multi-morbid disorders (Boyd, C. M., Darer, J., Boult, C., Fried, L.P., Boult, L., et al. (2005). Clinical Practice Guidelines and Quality of Care for Older Patients With Multiple Comorbid Diseases: Implications for Pay for Performance. *JAMA,* 294(6), 716–724.

2 Measures such as Qaly and Daly are generally viewed as ‘economic parameters’. Added with the complexity of capturing the impact on Qaly’s, these were not included in the evaluation of HVCCC projects. Nevertheless, the focus of these projects on quality of life and avoiding life years spent in poor quality health could have an impact on these parameters.
