# Supplementary material for: Doctors as Resource Stewards? Translating High-Value, Cost-Conscious Care to the Consulting Room
Source: Health Care Anal. 2022 May 13;30(3-4):215–39. doi: 10.1007/s10728-022-00446-4 (PMC9741564; doi:10.1007/s10728-022-00446-4)
Supplement: Supplementary file 1 — Supplementary file1 (PDF 86 kb) [file 10728_2022_446_MOESM1_ESM.pdf]

Appendix Interview guide

1. Could you tell me something about the project that you are currently working on?

- *What problem does your project address?*

- *What is the main goal of your project?*

- *Who are involved in your project / are supervisors involved? What is their level of involvement?*

2. How would you define high-value, cost-conscious care in your own words?

3. What are the envisioned goals of your project?

- *When do you consider your project to be successful?*

HVCCC Matrix

<Interviewer introduces and explains the HVCCC Matrix>

We developed a 'HVCCC matrix' based on a conceptualisation of high-value, cost-conscious care from a paper by Owens and colleagues. (2011), in order to be able to compare findings across different interviews.<br><br>The matrix distinguishes four dimensions of HVCCC:<br>- The promotion of good-value care delivery: in other words: projects that intend to promote the quality of care.<br>- Elimination of low-value care delivery: projects intend to reduce unnecessary care and other sources of waste.<br>- Reduction of healthcare costs: projects intend to lower costs of the care provided.<br>- Raising cost awareness: projects that intend to improve physicians' awareness of costs.

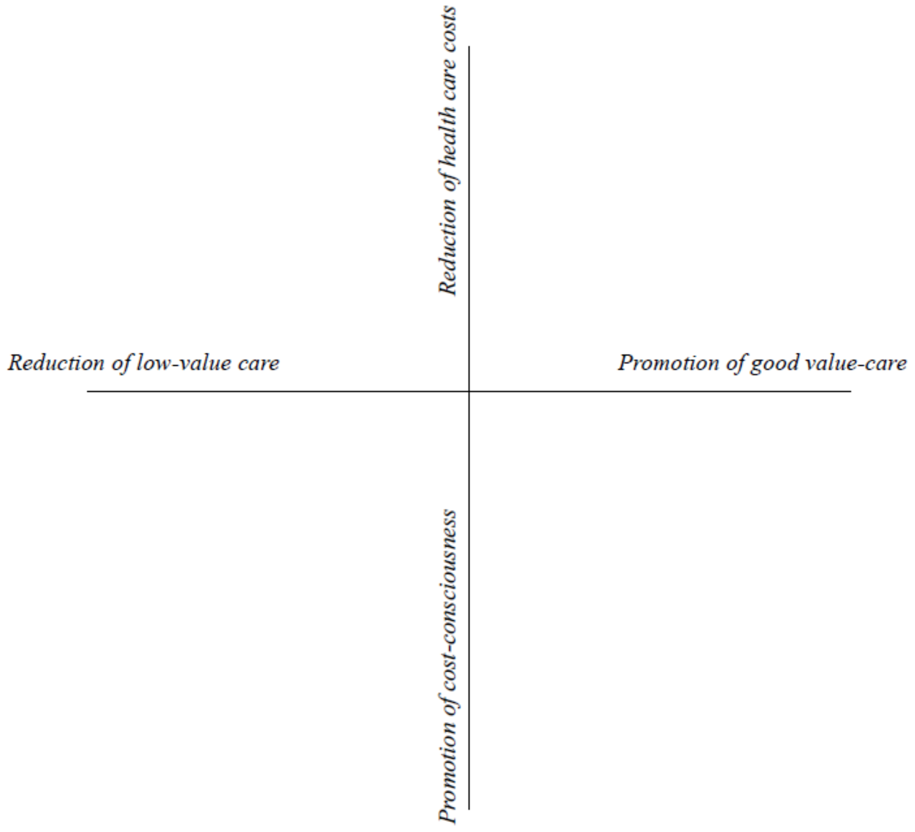

4. Could you plot the *envisioned project* goals on the matrix?

What facet(s) of high-value, cost-conscious care align with the envisioned goals of your project?

- Promotion of good-value care delivery
- Elimination of low-value care delivery
- Reduction of healthcare costs
- Raising cost awareness

Could you explain how the project could contribute to that? (what do they mean to you?)

HVCCC Outcomes

5. Could you tell us something about the actual outcomes of your project?

- *Are the outcomes different than expected?*

- *Are you expected to measure the outcomes of your project (e.g., in terms of cost savings? quality of care?)*

6. What factors affect(ed) your project outcomes?

- *How does [factor] affect your project exactly?*

- *What barriers have you encountered that made it more difficult to deliver HVCCC?*

* Questions in italic are probing questions. Depending on the interview, these or other probing questions could be used to unravel differences between espoused HVCCC and HVCCC in use.
